# Children’s privacy, autonomy, and digital identity in sharenting research: a scoping review with bibliometric mapping

**DOI:** 10.3389/fpsyg.2026.1845715

**Published:** 2026-06-11

**Authors:** Osman Akay

**Affiliations:** Department of Social Work, Istanbul Medipol University, Istanbul, Türkiye

**Keywords:** autonomy, children’s privacy, digital identity, digital parenting, online visibility, sharenting

## Abstract

**Introduction:**

Research on digital parenting and sharenting has expanded rapidly, yet the literature remains distributed across media studies, child development, communication, law, and health-related domains. This dispersal has limited integrative understanding of how parental online disclosure is linked to children’s privacy, autonomy, digital identity, and digitally mediated visibility.

**Methods:**

This study synthesized publications indexed in Web of Science Core Collection and Scopus at the intersection of digital parenting and sharenting. Guided by a structured review question, the study was conducted as a scoping review with bibliometric mapping and reported in line with PRISMA-ScR principles. The review aimed to map conceptual, thematic, and evidentiary patterns across an interdisciplinary corpus rather than to perform quality-weighted effect synthesis. Records were screened against four explicit eligibility criteria concerning children’s privacy, online visibility, digital identity, consent, and parent–child boundary regulation. The final corpus comprised 252 English-language articles and reviews published between 1992 and 2025. Narrative synthesis was combined with bibliometric mapping using bibliometrix in R and VOSviewer to identify thematic concentrations, intellectual linkages, and temporal trends.

**Results:**

The field showed limited early output, followed by sustained growth after 2016 and marked acceleration from 2019 onward. The literature has consolidated around a conceptual core defined by sharenting, social media, children, privacy, and digital parenting. Communication Privacy Management, privacy calculus, mindful sharenting, and psychological ownership emerged as recurrent interpretive frameworks. Across the corpus, children’s privacy, consent, boundary negotiation, and digital identity increasingly functioned as organizing concerns, while more recent studies showed stronger engagement with platformized visibility, influencer culture, and rights-based governance. Intellectual mapping indicated a coherent citation base anchored in foundational work on sharenting, adolescents’ perceptions, and children’s privacy.

**Conclusion:**

The review indicates that sharenting is no longer examined solely as an everyday parental sharing practice. It is increasingly treated as a psychologically and ethically consequential process through which children’s online identities are constructed, negotiated, and exposed in platformed environments. The study clarifies the conceptual and intellectual structure of the field and highlights the need for more child-centered, cross-cultural, and psychologically explicit research.

## 1 Introduction

Digital technologies have become deeply embedded in family life, transforming how parents guide, monitor, and shape children’s participation in networked environments. Within this context, digital parenting has emerged as a broad construct encompassing parental mediation, co-use, monitoring, and guidance strategies intended to support children’s digital experiences and wellbeing (Modecki et al., 2022; Tan et al., 2024). Yet digital parenting increasingly extends beyond regulating children’s screen use. It also involves the production, circulation, and management of children’s digital traces in social media and other platform-mediated environments. This shift is especially visible in sharenting, commonly understood as parents’ online sharing of photographs, videos, and personal information about their children (Blum-Ross and Livingstone, 2017; Brosch, 2016, 2018).

Early scholarship often framed sharenting as part of ordinary family documentation, identity work, and social support, emphasizing parents’ efforts to narrate everyday life, preserve memories, and maintain relational ties through online sharing (Kumar and Schoenebeck, 2015; Blum-Ross and Livingstone, 2017). Over time, however, sharenting came to be understood as more than a benign extension of parental self-expression. Research increasingly linked it to questions of privacy, overexposure, reputational risk, and the long-term persistence of digital traces, thereby placing sharenting at the intersection of parenting, media use, and children’s rights (Choi and Lewallen, 2018; Holiday et al., 2022). Child- and adolescent-centered studies further show that children may object to parental posting, experience privacy boundary turbulence, and perceive sharenting as a source of family conflict when consent and control are not negotiated (Lipu and Siibak, 2019; Garmendia et al., 2022).

A key gap in the existing literature concerns the lack of an integrated, field-level synthesis that maps how psychologically relevant constructs—privacy, consent, autonomy, and digital identity—are conceptually organized across this interdisciplinary body of work. Although children’s privacy and autonomy have become increasingly prominent themes, existing reviews have largely developed along parallel tracks: some have focused on digital parenting as a broad domain (Modecki et al., 2022; Tan et al., 2024), some on sharenting practices as such (Cino, 2021; Tosuuntaş and Griffiths, 2024), some on scientometric patterns (Cataldo et al., 2022), and others on children’s digital identity formation (Berg et al., 2024). This division has made it difficult to evaluate how these constructs are organized across the field as a whole.

A recurrent finding in the literature is that sharenting is characterized by ambivalence. Parents often report motivations such as memory keeping, emotional expression, pride, social connection, and access to support, while simultaneously acknowledging privacy concerns and potential harm (Cino, 2021; Cataldo et al., 2022; Tosuuntaş and Griffiths, 2024). This tension has been explored through several frameworks. Communication Privacy Management theory has been used to examine boundary negotiations between parents and children over online disclosure (Walrave et al., 2022). Mindful sharenting has highlighted reflective and selective sharing practices (Walrave et al., 2023). Privacy calculus and multiparty privacy perspectives explain why parents continue to share despite recognizing risk (Ní Bhroin et al., 2022; Peng, 2023, 2024), whereas psychological ownership addresses parents’ perceived authority over children’s information (Cai, 2023). Collectively, these approaches indicate that sharenting is shaped by the interaction of relational norms, moral reasoning, and platform affordances.

Legal and policy-oriented scholarship has further framed sharenting as a matter of privacy, dignity, best interests, and digital rights. Steinberg (2017) described sharenting as a conflict between parental expression and child protection, while Kravchuk (2021) and Lendvai (2024) showed that regulatory responses remain fragmented. Empirical work on parenting style, health disclosures, consumer vulnerability, influencer practices, and platform commercialization further suggests that the consequences of parental sharing may extend to surveillance, profiling, commercial engagement, and commodified visibility (Amon et al., 2022; Ong et al., 2022; Tartari et al., 2023; Vizcaíno-Verdú et al., 2023; Fox et al., 2023; Baxter and Czarnecka, 2025). This expansion has rendered the field increasingly interdisciplinary.

To address this gap, the present study was conducted as a scoping review with bibliometric mapping. The review asked how children’s privacy, autonomy, digital identity, online visibility, consent, and parent–child boundary regulation are conceptualized across the sharenting literature; which theoretical and methodological traditions shape the field; and what substantive gaps remain, particularly with respect to children’s own perspectives, developmental variation, and cross-cultural coverage. In line with these questions, the review examined the field’s temporal development, core concepts, recurring theoretical frameworks, thematic patterns, bibliometric structure, and remaining methodological and contextual gaps.

## 2 Methods

### 2.1 Design, review question, and scope

This study was designed as a scoping review with complementary bibliometric mapping. A scoping review design was selected because the primary aim was to map the conceptual and intellectual structure of a heterogeneous, interdisciplinary field rather than to synthesize effect-size estimates from intervention studies (Arksey and O’Malley, 2005; Peters et al., 2020). Scoping reviews are appropriate when the goal is to identify key concepts, clarify the boundaries of a literature, and detect gaps across diverse evidence types, including empirical, conceptual, legal, and review-based contributions (Tricco et al., 2018). The present corpus satisfies all three conditions. Formal risk-of-bias appraisal was therefore not applied; instead, methodological characteristics and evidentiary limitations were addressed descriptively. The review process was organized in accordance with the PRISMA extension for scoping reviews (PRISMA-ScR; Tricco et al., 2018). The reporting of the study selection flow was also informed by PRISMA 2020 reporting conventions (Page et al., 2021).

The overarching review question was formulated using the Population–Concept–Context (PCC) framework recommended for scoping reviews (Peters et al., 2020):

*Population*: Publications addressing children and adolescents in relation to parental online sharing practices (sharenting, digital parenting).
*Concept*: Children’s privacy, autonomy, digital identity, online visibility, consent, and parent–child boundary regulation in the context of parental online disclosure.
*Context*: English-language peer-reviewed articles and reviews indexed in Web of Science Core Collection and Scopus between 1992 and 2025.

The overarching research question reads: “What is the conceptual, intellectual, and thematic structure of the literature at the intersection of digital parenting and sharenting, and how are children’s privacy, autonomy, digital identity, and online visibility conceptualized, theorized, and examined across this interdisciplinary field?” Six subsidiary questions guided the analysis: (RQ1) How has the literature developed temporally? (RQ2) How are children’s privacy, autonomy, and digital identity conceptualized in the field? (RQ3) What psychological and relational frameworks recur in the literature? (RQ4) What thematic patterns characterize the corpus? (RQ5) What intellectual linkages and concentrations are visible through bibliometric mapping? (RQ6) What methodological and contextual gaps remain underaddressed?

Bibliometric techniques were used in a secondary and supportive capacity to identify broad thematic concentrations, intellectual linkages, and temporal trends rather than as the sole analytic basis of the study. The review was intended to map the scope and structure of the literature, not to estimate pooled effects or rank studies by methodological quality.

### 2.2 Data sources and search strategy

The literature search was conducted in the Web of Science Core Collection and Scopus on March 15, 2026. These databases were selected because of their multidisciplinary coverage and their widespread use in evidence synthesis and bibliometric research. The search strategy was developed to capture publications located at the intersection of digital parenting and sharenting, especially work addressing children’s privacy, digital footprints, online visibility, consent, digital identity, and related rights-based concerns.

In Web of Science, the search was conducted using the Topic field with the following query: ((sharenting OR “social media parenting” OR (parent NEAR/3 shar) OR “parent influencer*” OR “family vlog*” OR “parent blog*” OR (“digital parenting” AND (privacy OR “online privacy” OR “children’s online privacy” OR “digital footprint*” OR “digital identit*” OR “online visibility” OR consent OR “children’s rights” OR “digital rights”))) AND (child* OR children OR minor* OR infant* OR toddler* OR adolescent*) AND (privacy OR “online privacy” OR “children’s online privacy” OR “digital footprint*” OR “digital identit*” OR “online visibility” OR consent OR “children’s rights” OR “digital rights”)).

In Scopus, the equivalent intersectional query was applied using the TITLE-ABS-KEY field, restricted to articles and reviews published in English between 1992 and 2025. Both queries required records to satisfy all three intersectional conditions simultaneously (sharing behavior AND child/adolescent population AND privacy/identity/consent constructs), performing substantive relevance filtering at the retrieval stage prior to screening.

### 2.3 Eligibility criteria and study selection

Records were considered eligible if they explicitly addressed at least one of the following four domains:

- parental online sharing of children’s images, information, or experiences (including sharenting, family vlogging, parent blogging, or parent influencer content);
- children’s or adolescents’ perceptions of, or responses to, parental online sharing;
- psychological, relational, developmental, ethical, or rights-related implications of parental disclosure that bear on children’s privacy, autonomy, or digital identity; or
- conceptual or review-based contributions that directly engage with children’s digitally mediated visibility in family-mediated digital environments.

Records whose primary focus was platform design optimization, purely commercial marketing strategy, or technical legal analysis without any engagement with child-level or family-level psychological or relational outcomes were not incorporated into the narrative synthesis, even if retained in the bibliometric corpus. This distinction between the bibliometric corpus (all 252 records) and the narrative synthesis corpus (the subset substantively engaging the four eligibility domains) is maintained throughout the results and discussion.

The initial search identified 389 records (181 from Web of Science, 208 from Scopus). After database merging, 136 duplicate records were removed, yielding 253 unique publications. One record indexed in 2026 was excluded to maintain temporal consistency, leaving a final corpus of 252 publications.

**Figure 1:**
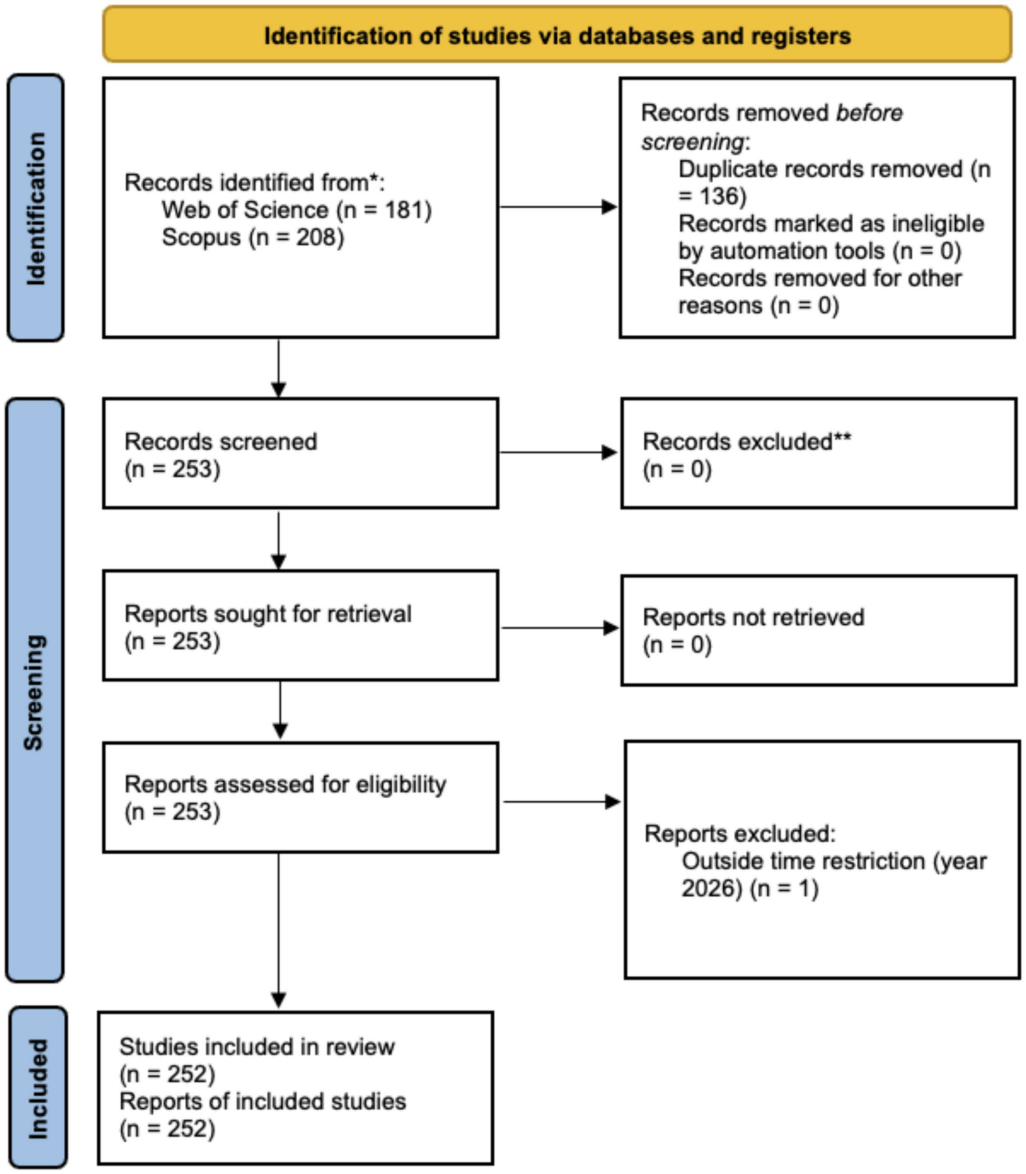
PRISMA-ScR flow diagram of study identification, deduplication, screening, and inclusion.

Title-and-abstract screening was conducted against the four eligibility criteria. Because the intersectional search strategy required each record to satisfy three conceptual conditions at retrieval (sharing behavior, child/adolescent population, and privacy/identity/consent concerns), screening primarily served to confirm conceptual fit rather than to remove large off-topic clusters. Full-text consultation was used for borderline cases, and the implications of this highly targeted retrieval strategy for selection transparency are acknowledged in the limitations. Figure 1 summarizes the identification, deduplication, screening, and inclusion process.

### 2.4 Data preparation, extraction, and synthesis

Bibliographic records were imported into R using the bibliometrix package and merged across databases. Cited-reference fields were restored and standardized by matching merged records to the original database exports through DOI- and title-based reconciliation procedures, followed by citation normalization. Cited-author and cited-source metadata were subsequently extracted to support cited-reference analyses (Aria and Cuccurullo, 2017).

For the purposes of narrative synthesis, studies were examined in relation to their conceptual orientation, focal constructs, and thematic relevance to children’s privacy, autonomy, digital identity, online visibility, parent–child boundary negotiation, and consent. The synthesis did not attempt to calculate pooled effects because the corpus consisted of interdisciplinary publications with heterogeneous designs, including empirical, conceptual, review, and governance-oriented contributions. The analytic emphasis was placed on identifying recurring themes, explanatory frameworks, and conceptual shifts across the field. Because the review was designed as a field-level scoping synthesis rather than an intervention-focused effect review, formal risk-of-bias assessment was not applied. Instead, methodological characteristics and recurring evidentiary limitations were addressed descriptively and integrated into the interpretation of gaps.

Following eligibility assessment, all 252 records were retained for bibliometric mapping, whereas a coded subset of 102 records substantively informed the narrative synthesis. Records were additionally coded on the basis of title, abstract, keywords, and source metadata with respect to perspective, evidence type, age group, focal construct, and broad regional location. This step was used to distinguish publications directly centered on sharenting and child-related disclosure issues from records that were relevant for field mapping but more peripheral to the interpretive synthesis.

### 2.5 Bibliometric mapping

Bibliometric mapping was used to support the synthesis by identifying areas of conceptual concentration, intellectual linkage, and temporal development. Descriptive indicators were calculated for annual scientific production, citation distribution, productive contributors, source concentration, and country-level participation. The conceptual structure of the field was examined through keyword co-occurrence and thematic evolution analyses based on authors’ keywords (DE). Thematic evolution was assessed across four periods (1992–2016, 2017–2021, 2022–2024, and 2025). The intellectual structure of the field was examined through cited-reference co-citation analysis. Network visualizations were generated using VOSviewer with association strength normalization and default clustering parameters (van Eck and Waltman, 2010).

### 2.6 Software

All data preparation, normalization, and bibliometric analyses were conducted in R using the bibliometrix package (Aria and Cuccurullo, 2017). Network visualizations were generated using VOSviewer (van Eck and Waltman, 2010).

## 3 Results

### 3.1 Study selection and corpus overview

The review corpus consisted of 252 publications indexed between 1992 and 2025, drawn from Web of Science and Scopus and retained after deduplication and relevance screening. The full corpus represents an interdisciplinary body of scholarship spanning communication, media studies, psychology, sociology, pediatrics, law, education, and consumer research. Although the broader literature includes legal, ethical, and governance-oriented analyses, the field as a whole is increasingly organized around psychologically salient concerns, particularly privacy management, consent, identity construction, parental mediation, and children’s responses to being represented online. Detailed productivity indicators, source concentration data, and author-level metrics are reported in Supplementary Tables S1–S6 and Supplementary Figures S1–S5.

For analytic clarity, the full bibliometric corpus (*n* = 252) was distinguished from the subset of records that substantively informed the narrative synthesis (*n* = 102). The remaining records were retained for bibliometric mapping because they contributed to the field’s citation structure, thematic development, or conceptual ecology, but they were not weighted equally in the interpretive synthesis. As shown in Supplementary Table S7, the narrative synthesis subset was dominated by parent-only empirical studies (58.8%), followed by legal/policy contributions (22.5%). Review articles accounted for 6.9%, whereas direct child- or adolescent-centered empirical studies remained limited. Supplementary Table S8 further shows that privacy was the most consistently represented construct across all contribution types, while autonomy, consent, and boundary negotiation appeared more unevenly across the evidence base.

### 3.2 Temporal development of the literature

**Figure 2:**
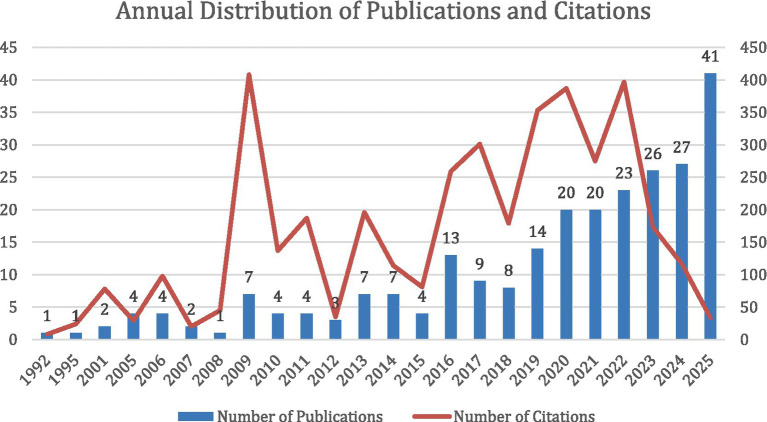
Annual distribution of publications and citations in the digital parenting–sharenting literature (1992–2025).

Publication output remained limited and irregular in the earlier years of the dataset, followed by a sustained increase after 2016 and a more pronounced acceleration from 2019 onward. The number of publications increased from 14 in 2019 to 20 in 2020 and remained at 20 in 2021, before rising to 23 in 2022, 26 in 2023, 27 in 2024, and 41 in 2025. Figure 2 shows the annual distribution of publications and citations.

This temporal pattern suggests that the field has moved from sporadic discussion to sustained scholarly consolidation. The acceleration aligns with the increasing visibility of platformized parenting practices, wider public debate on children’s digital privacy, and the expansion of influencer and family content economies. Citation counts fluctuate across the study period and show peaks in selected years, indicating a field that is both rapidly growing and increasingly anchored in a relatively stable body of widely cited reference points.

### 3.3 Children’s and adolescents’ perspectives in the evidence base

Direct child- and adolescent-centered evidence remained limited within the narrative synthesis subset. Of the 102 records coded as substantively informing the narrative synthesis, only 8 studies (7.8%) directly incorporated children’s or adolescents’ own perspectives, including 5 child/adolescent-only empirical studies and 3 mixed parent–child studies. By contrast, 60 studies (58.8%) relied on parent-only empirical evidence, and a further 34 records consisted of legal/policy, review, or conceptual/theoretical contributions (Supplementary Table S7).

Age representation was also highly uneven. Most direct evidence clustered around adolescence, with 6 studies focused primarily on participants aged approximately 14–17 years, 1 study centered on pre-teens aged roughly 10–13 years, and only 1 study spanning a broader child-to-adolescent range. No substantial body of direct evidence from younger children emerged in the coded subset. Methodologically, the child-centered evidence that does exist is relatively narrow and is concentrated mainly in qualitative or small-scale empirical work rather than in a broader and developmentally stratified evidence base.

Taken together, these patterns indicate that children’s privacy and autonomy are conceptually prominent in the literature, but the empirical evidence base remains disproportionately adult-defined and developmentally narrow. This imbalance is also visible in the distribution of focal constructs across contribution types. As shown in Supplementary Table S8, privacy is widely represented across parent-focused empirical, legal/policy, and review-based work, whereas autonomy, consent, and digital identity are comparatively less often examined through studies centered directly on children’s own accounts.

This imbalance is particularly evident for autonomy. Although autonomy is frequently invoked as a core concern in the sharenting literature, Supplementary Table S8 shows that it appeared in only 10 records across the coded narrative synthesis subset, indicating that its empirical representation remains limited relative to its conceptual prominence.

### 3.4 Conceptual organization of the field

**Figure 3:**
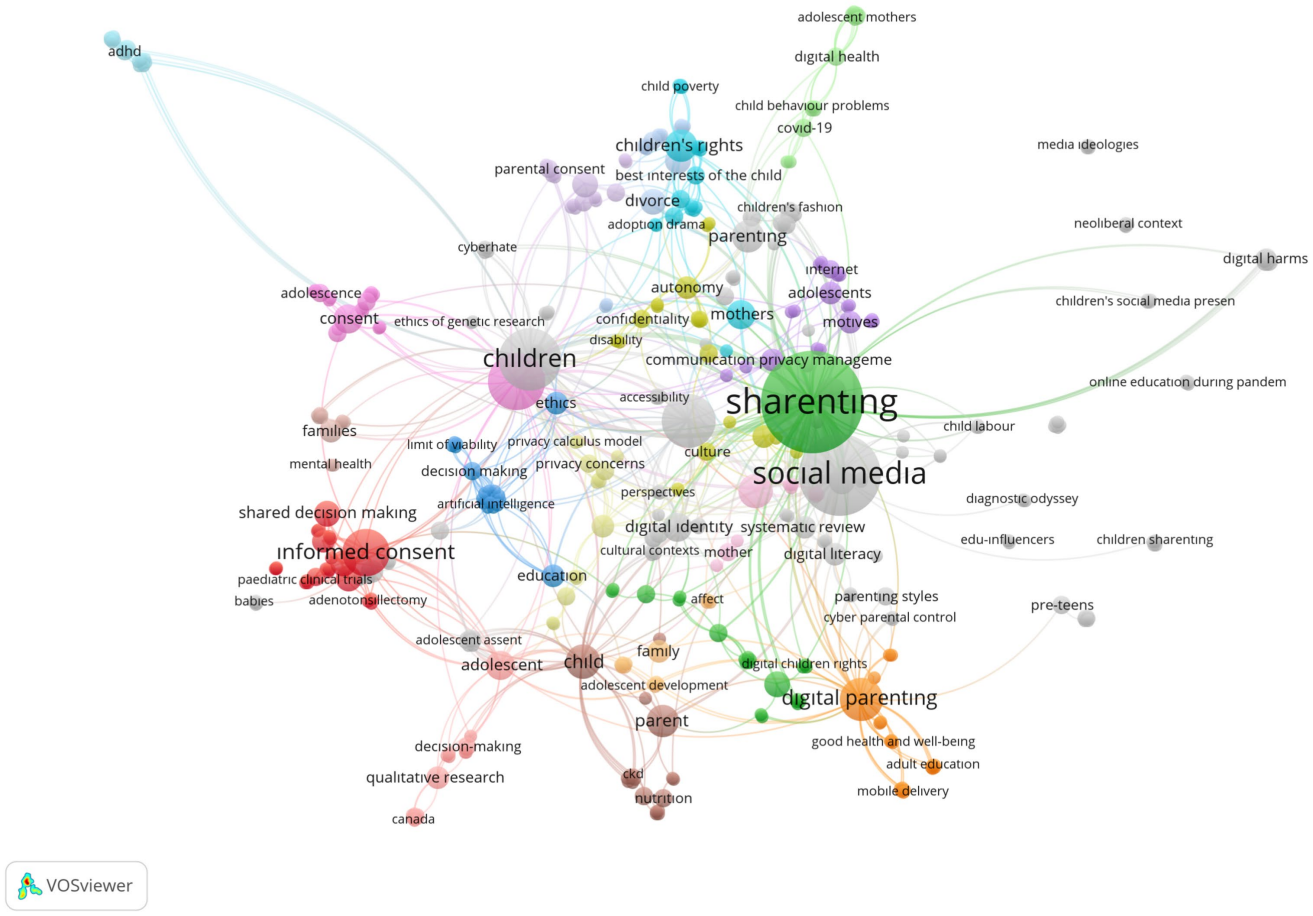
Keyword co-occurrence network in the digital parenting–sharenting literature.

Across the reviewed corpus, the literature converged around a recognizable conceptual core centered on sharenting, social media, children, privacy, and digital parenting. This pattern is visible in the keyword co-occurrence map presented in Figure 3, where these terms occupy the largest and most central positions in the network. Their prominence indicates that the field is no longer organized merely around parental media use, but around the consequences of parental disclosure for children’s digital presence and boundary management.

A second layer of concepts clusters around children’s rights, informed consent, parenting, adolescents, communication privacy management, and digital identity. These terms suggest that the field increasingly interprets sharenting through the language of relational negotiation, rights, visibility, and representation rather than through a narrow focus on posting behavior alone. Smaller peripheral terms, including digital harms, child labour, ADHD, pre-teens, and pandemic-related online education, indicate that the field also branches into adjacent concerns when parental disclosure intersects with vulnerability, commercialization, health, or crisis-mediated media practices.

### 3.5 Psychological and relational frameworks in the literature

The corpus draws on several recurring frameworks to explain why parents share, how disclosure boundaries are negotiated, and why children may experience parental posting as protective, relationally meaningful, or intrusive. Communication Privacy Management theory appears as one of the most visible interpretive frameworks, especially in work examining how parents and adolescents negotiate acceptable boundaries around disclosure (Walrave et al., 2022). This orientation treats online sharing as a form of boundary management rather than a purely individual preference.

A related strand conceptualizes selective online disclosure through mindful sharenting, emphasizing reflective decision-making, restraint, and balance between social connectedness and privacy protection (Walrave et al., 2023). Privacy calculus and multiparty privacy approaches explain why parents continue to disclose even when they recognize privacy risks, highlighting perceived social benefit, normative pressure, and relational reward as counterweights to concern (Ní Bhroin et al., 2022; Peng, 2023, 2024). Psychological ownership extends this discussion by showing how parents may perceive children’s personal information as partly under parental stewardship, thereby naturalizing sharing decisions that children themselves may later contest (Cai, 2023).

Taken together, these frameworks suggest that sharenting research is increasingly shaped by questions of agency, control, consent, and relational authority. The field has therefore moved closer to concerns that are central to developmental, social, and media psychology, especially where children’s emerging selfhood intersects with the communicative practices of caregivers.

### 3.6 Intellectual and thematic structure

**Figure 4:**
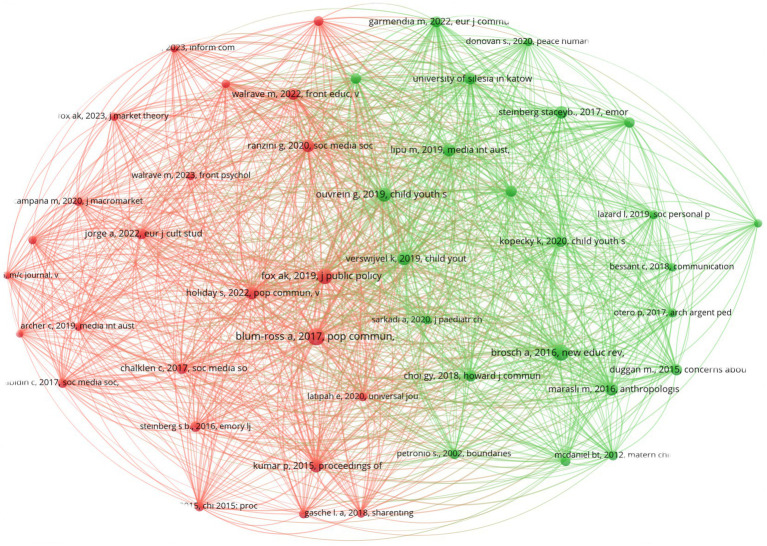
Cited-reference co-citation network in the digital parenting–sharenting literature.

The cited-reference co-citation network presented in Figure 4 reveals an intellectual structure organized around a relatively coherent set of foundational references. Frequently co-cited works include Blum-Ross and Livingstone (2017), Brosch (2016), Steinberg (2017), Ouvrein and Verswijvel (2019), Verswijvel et al. (2019), and Fox and Hoy (2019). These works collectively establish the field’s definitional, normative, and empirical foundations by linking parental sharing to privacy, adolescents’ perceptions, online self-presentation, and children’s rights.

**Figure 5:**
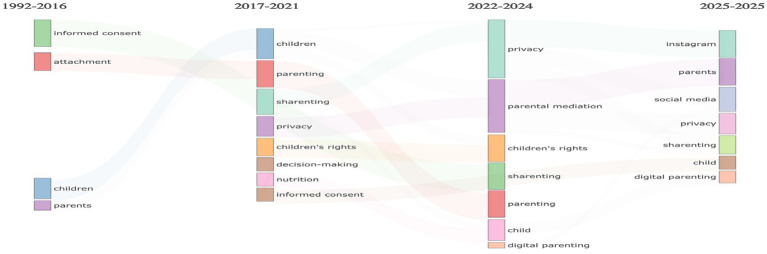
Thematic evolution in the digital parenting–sharenting literature.

Thematic evolution analysis further indicates that the field has become progressively more specific and psychologically focused over time (Figure 5). In the earliest period (1992–2016), themes such as informed consent, attachment, children, and parents were more prominent, reflecting roots in pediatric and developmental discussions. During 2017–2021, privacy, children’s rights, decision-making, and sharenting became more central, showing the field’s turn toward social media and rights-based debate. In 2022–2024, privacy, parental mediation, children’s rights, sharenting, and digital parenting formed a more consolidated thematic cluster. In 2025, Instagram, social media, privacy, sharenting, and digital parenting became especially prominent, pointing to a stronger platform-specific orientation and greater attention to visual sharing cultures and influencer practices.

### 3.7 Geographic distribution and collaboration patterns

The literature is geographically concentrated. The United States contributed the largest number of publications (56), followed by the United Kingdom (21), Canada (20), Italy (13), and Belgium (11). Corresponding-author data further indicate that the United States is the leading country in both overall output and single-country production, while Belgium shows a comparatively stronger multiple-country collaboration profile. Türkiye appears in the corpus with eight publications, all classified as single-country contributions.

International collaboration is present but uneven. The country collaboration network shows the United States in a structurally central position, connected to the United Kingdom, Canada, Australia, Italy, Belgium, Spain, and several other countries. At the author level, collaboration is organized into multiple clusters of varying size rather than a single integrated network. Overall, the field appears collaborative in parts, but still marked by regional concentration and uneven transnational integration.

### 3.8 Methodological and contextual gaps

The review points to several recurring gaps. First, the field remains concentrated in a limited set of countries and publication venues, which may shape how privacy, autonomy, and parental authority are conceptualized. The dominance of output from the United States, the United Kingdom, Canada, and a small number of European countries suggests that prevailing models may reflect culturally specific assumptions about family boundaries, platform use, and rights discourse.

Second, although children’s privacy and autonomy are prominent themes, the literature continues to rely heavily on parental perspectives, conceptual analyses, and governance-oriented concerns. Children’s and adolescents’ own perspectives are increasingly visible, but they do not yet occupy the same centrality as parental motivations and adult-defined privacy frameworks. Third, the more recent thematic turn toward Instagram, influencer cultures, and platformized visibility suggests that the field is becoming more sensitive to commercial and reputational dimensions of disclosure, yet longitudinal evidence on psychological outcomes remains comparatively limited. These patterns indicate a need for more child-centered, cross-cultural, and longitudinal work that examines how online representation affects children across developmental stages and social contexts.

## 4 Discussion

The discussion below does not treat conceptual prominence in the literature as equivalent to empirical maturity. The following discussion integrates the bibliometric and narrative findings to offer a critical interpretation of the field’s development. It is important to note at the outset that the corpus encompasses empirical, conceptual, legal, and governance-oriented publications. Where interpretive claims are grounded in empirical findings, they are explicitly attributed to specific types of evidence (e.g., survey-based, interview-based). Where claims remain primarily conceptual or normative, this is signaled accordingly. Gaps between conceptual prominence and available empirical evidence are noted rather than glossed over.

### 4.1 From documentation to digital personhood

The review demonstrates that research on digital parenting and sharenting has moved from a peripheral and fragmented area of inquiry to a more coherent interdisciplinary field with a distinct psychological core. Although scholarship still spans multiple disciplines, the field is increasingly organized around issues directly relevant to psychology: privacy management, autonomy, embarrassment, identity construction, consent, and relational boundary negotiation. This shift is visible not only in the thematic structure of the literature, but also in the way frequently cited and conceptually central works have defined the stakes of parental online disclosure.

One of the clearest patterns in the corpus is the transition from viewing sharenting as a routine form of family documentation to understanding it as a process of digitally mediated identity construction. Earlier work often focused on parental self-presentation, memory keeping, and social support, whereas later studies more explicitly interrogated the implications of parental posting for children’s dignity, privacy, and future self-determination. This development is theoretically important because it reframes children not merely as passive subjects of family storytelling, but as persons whose online representation may be shaped in advance of their ability to meaningfully authorize, contest, or reinterpret that representation. It should be noted, however, that this reframing remains primarily a conceptual and normative proposition in the literature; longitudinal empirical evidence demonstrating that early sharenting causes lasting harm to children’s sense of digital self-authorship is, as yet, limited.

### 4.2 Frameworks, persistence, and contestability

The prominence of Communication Privacy Management theory, privacy calculus, mindful sharenting, and psychological ownership shows that the field is converging on models capable of explaining both the persistence and the contestability of parental sharing. Survey-based and interview-based research using these frameworks consistently finds that parents may continue to disclose despite explicit privacy concerns, that they often view their decisions as protective or benign, and that children may nonetheless experience those same disclosures as intrusive, misaligned, or humiliating (Walrave et al., 2022; Walrave et al., 2023; Peng, 2023, 2024; Cai, 2023). Importantly, these frameworks also suggest that sharenting cannot be reduced to a simple opposition between responsible and irresponsible parenting. It is better understood as a site of ongoing negotiation in which parental intention, child subjectivity, relational norms, and platform logic intersect.

The bibliometric findings reinforce this interpretation. The keyword network places privacy, children, social media, digital parenting, and sharenting at the center of the field, while the co-citation map shows that definitional, empirical, and normative studies are increasingly cited together. This pattern indicates that the literature is developing a shared conceptual vocabulary. At the same time, the thematic evolution results suggest that the field is becoming more platform-specific and more attuned to the governance implications of online visibility, yet, as the following section details, the conceptual vocabulary consolidated around ‘autonomy’ and ‘children’s rights’ often outpaces the empirical evidence centered on children’s own developmental experiences.

### 4.3 Children’s perspectives, autonomy, and the limits of adult-defined privacy frameworks

A substantive imbalance between the conceptual centrality of children’s privacy and autonomy and the actual volume of child-centered empirical evidence runs through the literature. Although autonomy and privacy appear prominently in keyword analyses and in the normative framing of many studies, the empirical base for these concepts is built predominantly on parental perspectives, adult-defined frameworks, and qualitative studies with adolescent samples. This imbalance was visible not only conceptually but also in the composition of the coded narrative synthesis subset, in which direct child- or adolescent-centered evidence accounted for only 7.8% of records, while parent-only empirical, legal/policy, and review-oriented contributions dominated the field (Supplementary Table S7).

Where children’s and adolescents’ own perspectives have been studied empirically, findings are nuanced. Qualitative and interview-based research shows that adolescents’ responses to parental sharing are highly variable: some tolerate or welcome selected forms of disclosure, whereas others actively resist posts they perceive as embarrassing, excessive, or beyond their control. However, this body of evidence remains concentrated primarily in adolescence. In the coded narrative synthesis subset, almost all direct child-centered evidence came from studies of adolescents or older pre-teens, with no substantial body of work centered on younger children. As a result, the literature remains limited in its ability to speak developmentally about how children at different ages understand privacy, consent, and visibility.

Although autonomy is frequently cited as a core concern of the field, the metadata-coded narrative synthesis subset (*n* = 102) indicates that it appeared as a focal construct in fewer than one in 10 records (*n* = 10; Supplementary Table S8), compared with privacy, which was coded in the large majority of records (*n* = 95). This gap between conceptual prominence and empirical operationalization warrants attention: a field that positions children’s autonomy as ethically central must also develop research designs capable of operationalizing and examining it directly. At present, autonomy appears to function more as a normative and interpretive anchor than as a comparably mature empirical domain.

The concept of consent is frequently invoked in the literature, particularly in legal and normative scholarship, as a frame for evaluating the ethical legitimacy of parental sharing. Yet empirical evidence on how consent capacity develops across age groups—and how children’s tolerance for and resistance to disclosure changes as they mature into adolescence—remains limited. The literature largely treats consent as a binary and age-independent threshold rather than as a developmentally graded capacity. This is a meaningful gap: if child-centered research is to move beyond noting that children’s perspectives are underrepresented, it needs to engage more explicitly with developmental stage as an analytic variable.

The geographic concentration of the literature compounds this limitation. Privacy expectations, family norms, children’s rights traditions, and everyday media practices vary considerably across cultural contexts. A field that aspires to speak about children’s autonomy and digital identity more broadly requires wider cross-cultural coverage and stronger participation from underrepresented regions, particularly contexts in which family visibility, interdependence, and digital participation are structured differently from the assumptions embedded in Anglo-European privacy discourse.

Accordingly, the current literature should be interpreted as conceptually rich but evidentially uneven. Privacy, autonomy, digital identity, and online visibility are well established as organizing concerns in the field, but they are not yet supported by an equally mature body of child-centered, developmentally differentiated, and cross-culturally distributed empirical research. This gap marks one of the clearest priorities for the next stage of sharenting research.

### 4.4 Implications for child-centered and cross-cultural research

The findings indicate several concrete directions for future research. First, empirical work should more consistently center children’s own accounts. This requires research designs that go beyond asking parents about their children’s perceived reactions, including participatory methodologies, child-inclusive interview protocols, and age-appropriate data collection instruments adapted for younger age groups. Second, longitudinal designs are needed to examine how the psychosocial consequences of sharenting—including effects on self-concept, relational trust, and privacy attitudes—develop as children who have been extensively shared about enter adolescence and early adulthood. Cross-sectional studies of adolescents cannot substitute for this developmental evidence.

Third, future research should adopt a more explicit developmental framing. Studies should distinguish between age groups rather than treating “children and adolescents” as a homogeneous category. The privacy concerns, autonomy expectations, and relational dynamics of a six-year-old are categorically different from those of a 15-year-old, and theory and research designs should reflect this. Fourth, cross-cultural and cross-regional studies are urgently needed to test whether the frameworks built within Anglo-European contexts—CPM theory, privacy calculus, mindful sharenting—generalize across different cultural configurations of family life, digital participation, and children’s rights. Fifth, as sharenting increasingly intersects with influencer economies and commercial platformization, research should examine the psychosocial consequences for children whose online identities are not only shared but monetized, building on existing foundational work in this area (Jorge et al., 2022; den Van Abeele et al., 2024, 2025; Beuckels et al., 2025).

### 4.5 Limitations

Several limitations should be considered when interpreting the findings. First, the corpus was restricted to English-language articles and reviews indexed in Web of Science and Scopus. Although this strategy ensured coverage of widely visible peer-reviewed literature, it may have excluded relevant work published in other languages, regional journals, books, or grey literature. Second, the review synthesized an interdisciplinary corpus containing empirical, conceptual, legal, and review-based publications. This breadth was important for mapping the field, but it also limited the feasibility of formal risk-of-bias appraisal and quantitative synthesis across studies.

Third, the study was conducted by a single author. The absence of a second independent screener means that the reproducibility of screening decisions cannot be verified and selection bias cannot be entirely ruled out. To partially compensate, eligibility criteria were operationalized prior to screening, and borderline cases were resolved by consulting full texts. Future replications of this review should incorporate dual independent screening to improve reproducibility. Fourth, the highly targeted intersectional search strategy reduced the number of clearly off-topic records retrieved and thus limited exclusions during screening. While this improved topical precision, it may also make the selection process appear less transparent than in conventional review workflows with larger exclusion counts. While this outcome is explained by the precision of the query rather than by absence of screening, it may nonetheless limit the transparency of the selection process for readers accustomed to conventional systematic reviews with explicit exclusion tallies.

Fifth, the search strategy was intentionally broad in order to capture the intersection of digital parenting and sharenting; as with all field-level reviews, relevant records may have been missed if they used substantially different terminology, and some included records may have engaged the core topic only indirectly. Finally, citation-based indicators are shaped by publication age, disciplinary citation norms, and database indexing practices, so bibliometric prominence should not be interpreted as a direct proxy for theoretical or methodological quality.

## 5 Conclusion

This study provides an integrated scoping review of research on digital parenting and sharenting with particular emphasis on children’s privacy, autonomy, digital identity, and online visibility. The review maps a field that is conceptually consolidating, yet still evidentially uneven. The findings show that the field has expanded rapidly and now exhibits a recognizable conceptual and intellectual core organized around sharenting, privacy, children, social media, and digital parenting. Recurrent psychological and relational frameworks—especially those concerned with privacy management, consent, ownership, and boundary regulation—have become central to explaining parental online disclosure and its consequences.

More broadly, the review indicates that sharenting should not be treated only as an everyday parental media practice. It is increasingly understood as a process through which children’s digital presence is created, negotiated, and potentially contested across developmental, relational, and platformized contexts. By integrating narrative synthesis with bibliometric mapping, the study clarifies how the field is structured, where it has become conceptually coherent, and where important gaps remain. Chief among these gaps are the underrepresentation of children’s own perspectives across age groups, the limited cross-cultural scope of the literature, and the absence of longitudinal evidence on the psychosocial consequences of parental online disclosure. Future research will benefit from stronger inclusion of children’s perspectives, wider cross-cultural scope, developmental explicitness, and more explicit attention to the long-term psychosocial implications of family-mediated digital visibility.

## Data Availability

The original contributions presented in the study are included in the article/Supplementary material, further inquiries can be directed to the corresponding author.
