# Supplementary material for: Children’s privacy, autonomy, and digital identity in sharenting research: a scoping review with bibliometric mapping
Source: Front Psychol. 2026 Jun 11;17:1845715. doi: 10.3389/fpsyg.2026.1845715 (PMC13293831; doi:10.3389/fpsyg.2026.1845715)
Supplement: Supplementary file 1 [file Supplementary_file_1.docx]

Supplementary material

Children’s Privacy, Autonomy, and Digital Identity in Sharenting Research: Supplementary Material


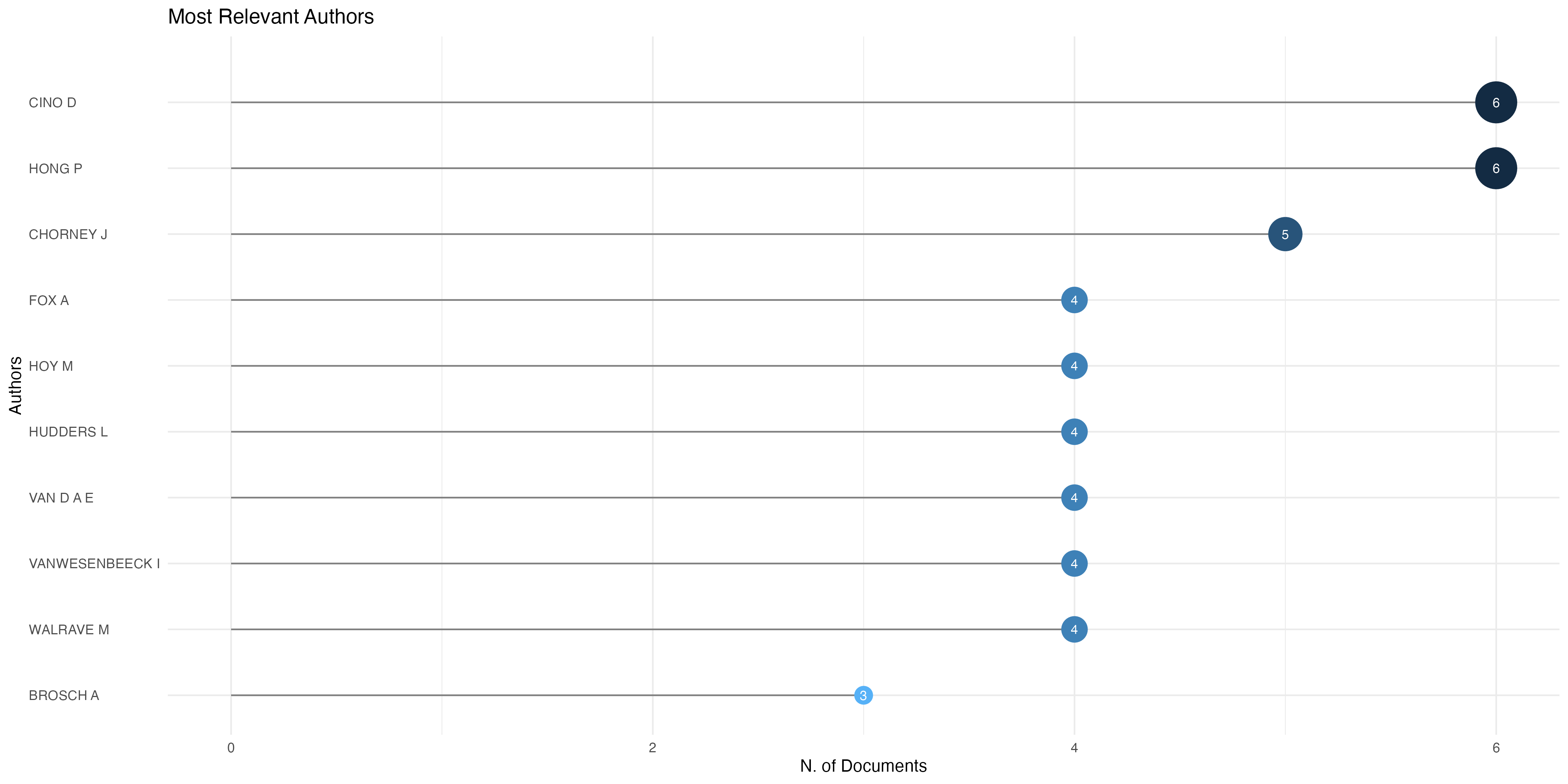


Figure S1.Most productive authors in the digital parenting–sharenting literature


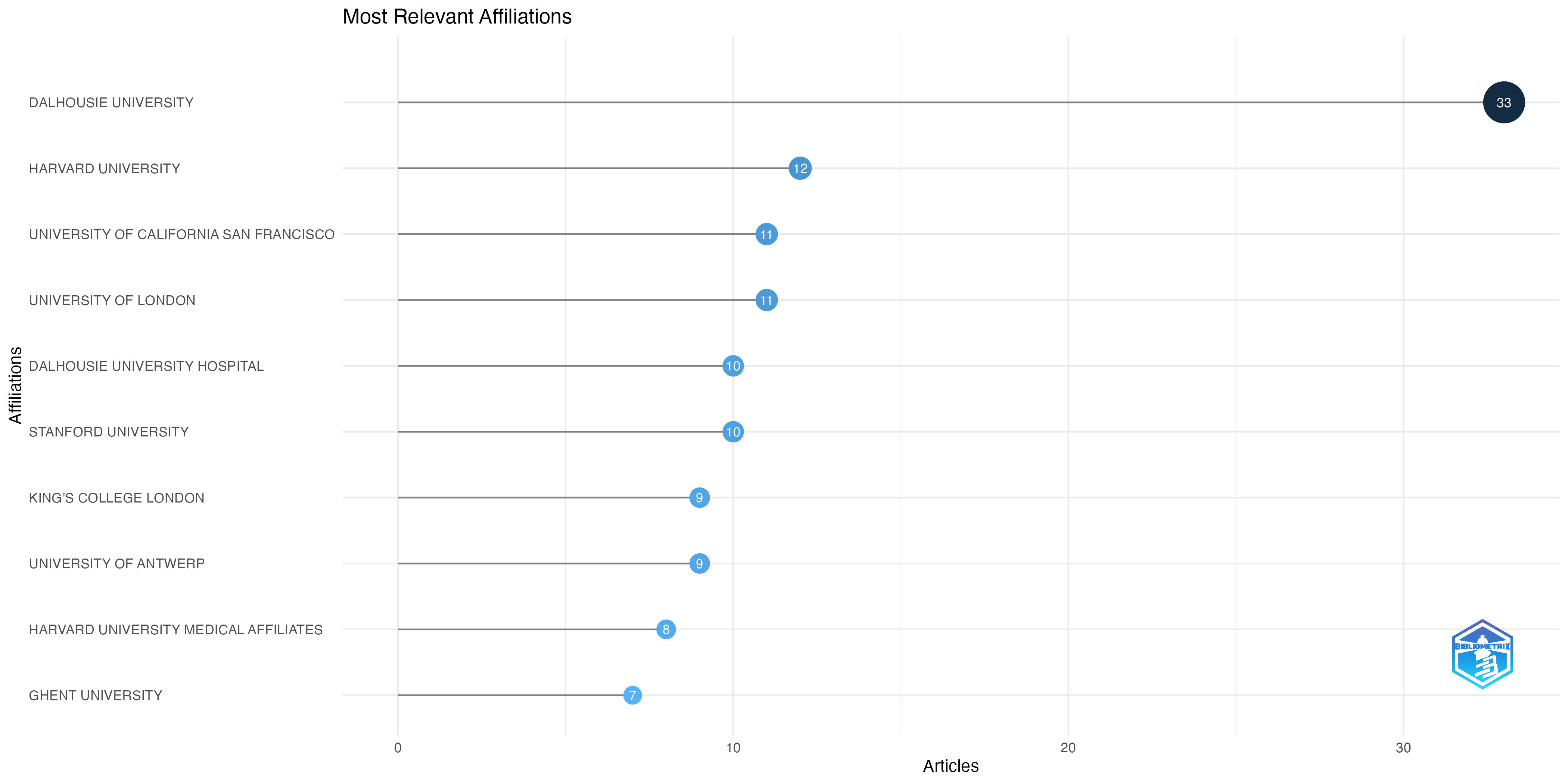


Figure S2. Most productive affiliations in the digital parenting–sharenting literature


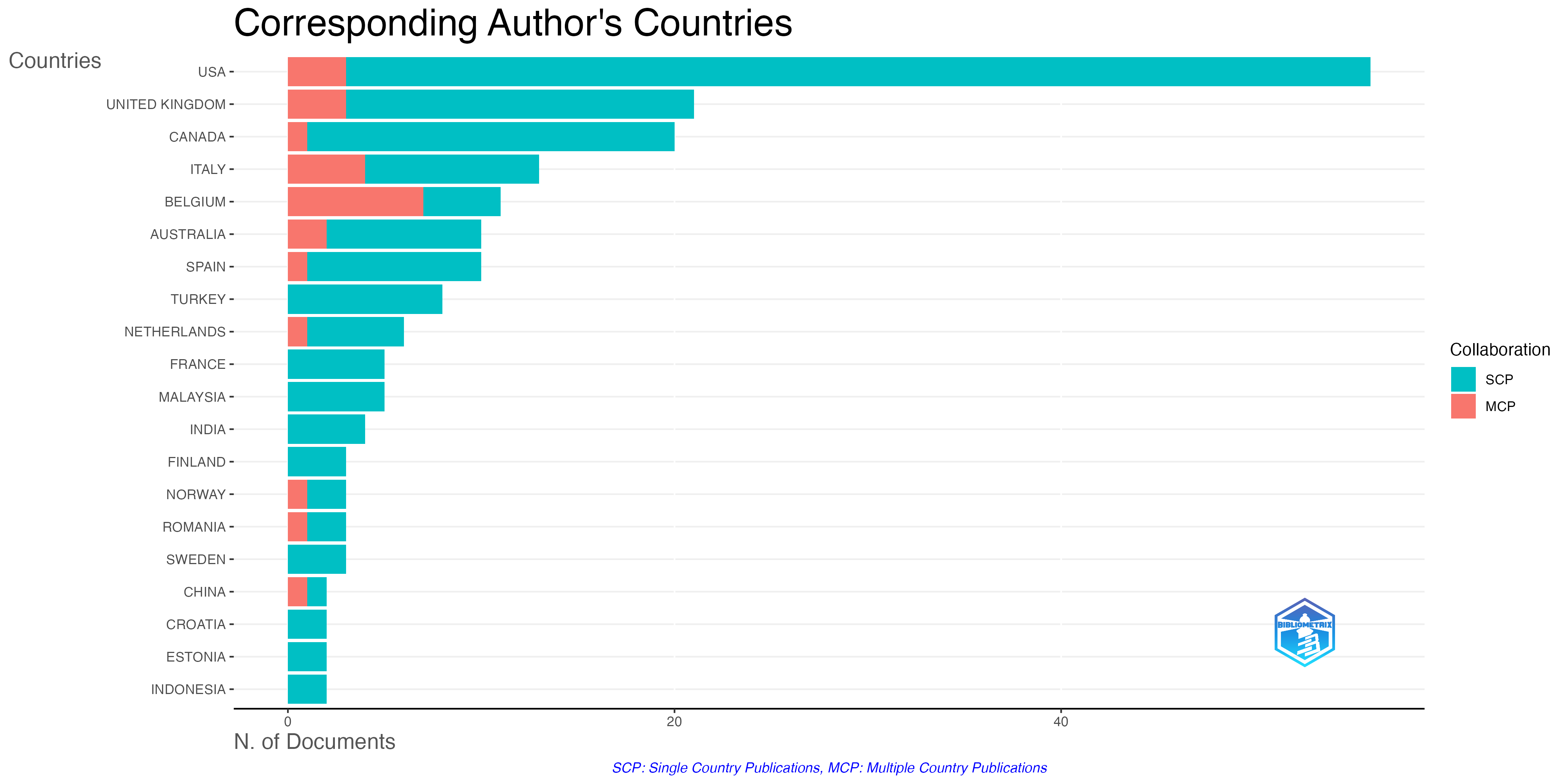


Figure S3. Corresponding author’s countries in the digital parenting–sharenting literature


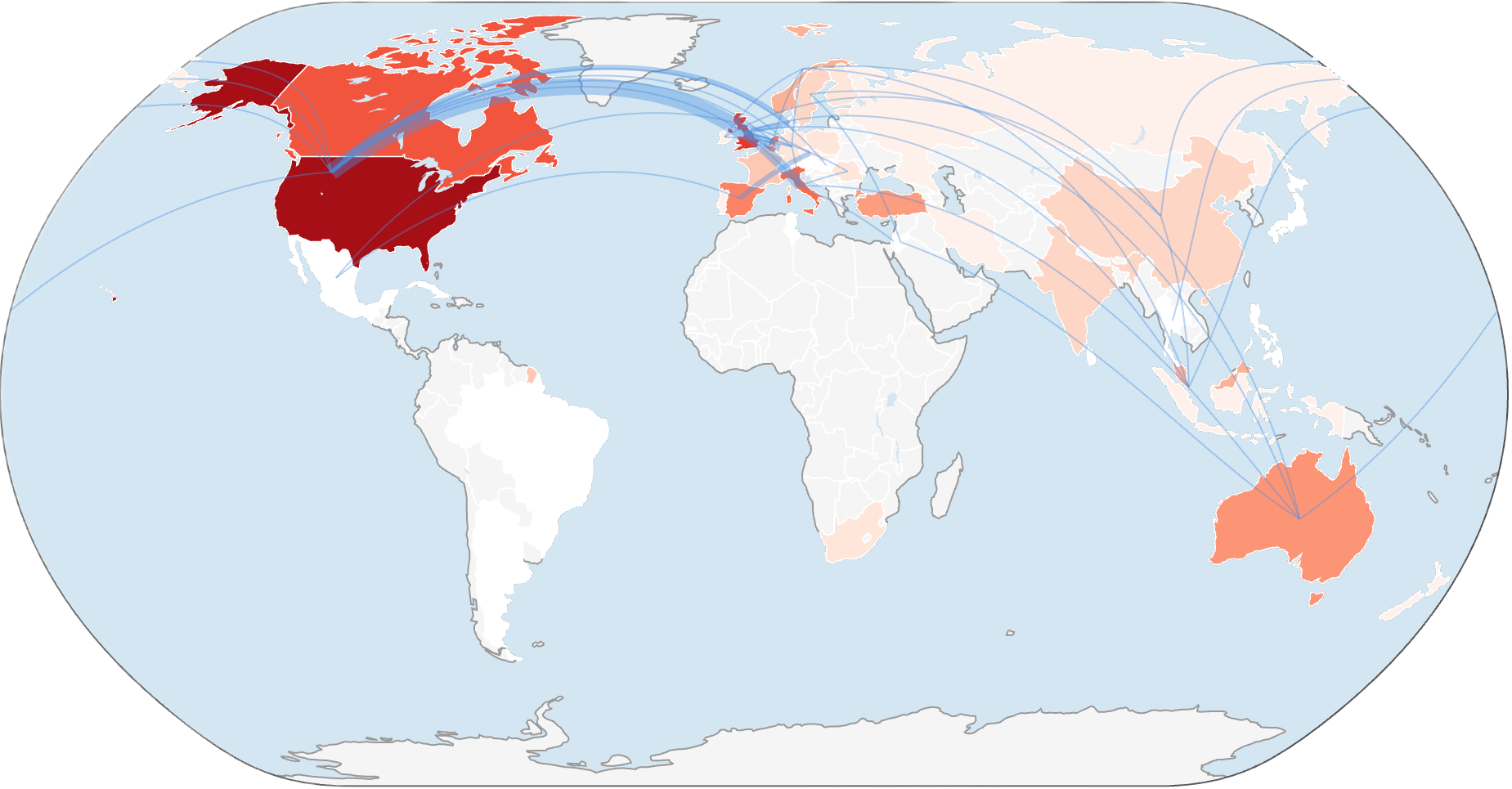


Figure S4. International collaboration network in the digital parenting–sharenting literature


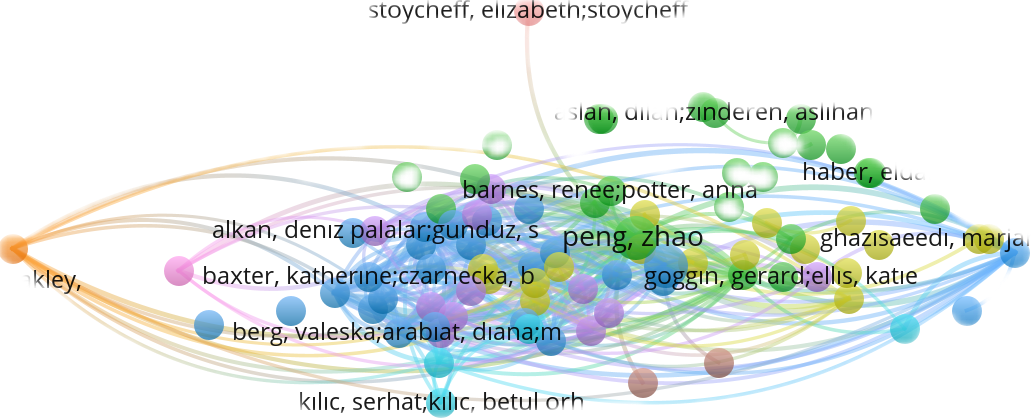


Figure S5. Co-authorship network of authors in the digital parenting–sharenting literature

**Table S1. Top 10 most cited publications in the digital parenting–sharenting literature**

| **Authors** | **Year** | **Article Title** | **Journal** | **Total Citations** |
| --- | --- | --- | --- | --- |
| Brosch, A. | 2016 | When the Child Is Born into the Internet: Sharenting as a Growing Trend Among Parents on Facebook | New Educational Review | 102 |
| Fox, A., et al. | 2019 | Smart Devices, Smart Decisions? Implications of Parents' Sharenting for Children's Online Privacy | Journal of Public Policy & Marketing | 90 |
| Holiday, S., et al. | 2022 | Sharenting and the Extended Self: Self-representation in Parents' Instagram Presentations of Their Children | Popular Communication | 69 |
| Verswijvel, K., et al. | 2019 | Sharenting: Adolescents’ Perceptions on Social Network Sites | Children and Youth Services Review | 65 |
| Modecki, K., et al. | 2022 | What Is Digital Parenting? A Systematic Review | Perspectives on Psychological Science | 61 |
| Ranzini, G., et al. | 2020 | Sharenting, Peer Influence, and Privacy Concerns on Instagram | Social Media + Society | 56 |
| Heilferty, C. | 2011 | Ethical Considerations in Online Illness Narratives | Journal of Advanced Nursing | 53 |
| Lipu, M., et al. | 2019 | “Take It Down!”: Parents’ and Pre-teens’ Opinions on Sharenting | Media International Australia | 49 |
| Brosch, A. | 2018 | Sharenting – Why Do Parents Violate Their Children’s Privacy? | New Educational Review | 49 |
| Barnes, R., et al. | 2021 | Sharenting and Parents' Digital Literacy | Communication Research and Practice | 46 |

**Note.** Total citations (TC) are based on merged citation data from Web of Science and Scopus.

**Table S2. Most productive authors, sources, and countries in the digital parenting–sharenting literature**

| **Category** | **Rank** | **Entity** | **Publications** |
| --- | --- | --- | --- |
| Author | 1 | Cino D | 6 |
| Author | 2 | Hong P | 6 |
| Author | 3 | Chorney J | 5 |
| Author | 4 | Fox A | 4 |
| Author | 5 | Hoy M | 4 |
| Source | 1 | Pediatrics | 6 |
| Source | 2 | Information, Communication & Society | 5 |
| Source | 3 | PLOS ONE | 4 |
| Source | 4 | Children and Youth Services Review | 3 |
| Source | 5 | Communication Research and Practice | 3 |
| Country | 1 | United States | 56 |
| Country | 2 | United Kingdom | 21 |
| Country | 3 | Canada | 20 |
| Country | 4 | Italy | 13 |
| Country | 5 | Belgium | 11 |

**Table S3. Author productivity distribution in the digital parenting–sharenting literature**

| **Documents** | **Number of Authors** | **Percentage (%)** |
| --- | --- | --- |
| 1 | 740 | 89.7 |
| 2 | 64 | 7.8 |
| 3 | 12 | 1.5 |
| 4 | 6 | 0.7 |
| 5 | 1 | 0.1 |
| 6 | 2 | 0.2 |
| Total | 825 | 100.0 |

***Note.*** Percentages are calculated based on the total number of unique authors in the corpus (N = 825).

**Table S4. Bradford zone distribution in the digital parenting–sharenting literature**

| **Zone** | **Journals** | **Articles** |
| --- | --- | --- |
| Zone 1 | 42 | 84 |
| Zone 2 | 84 | 84 |
| Zone 3 | 84 | 84 |

***Note.*** Zone 1 represents the core journals, whereas Zones 2 and 3 represent progressively more peripheral journal groups.

**Table S5. Core journals in Zone 1 by number of publications**

| **Rank** | **Journal** | **Articles** |
| --- | --- | --- |
| 1 | Pediatrics | 6 |
| 2 | Information, Communication & Society | 5 |
| 3 | PLOS ONE | 4 |
| 4 | Children and Youth Services Review | 3 |
| 5 | Communication Research and Practice | 3 |
| 6 | International Journal of Children’s Rights | 3 |
| 7 | New Educational Review | 3 |
| 8 | Proceedings of the ACM on Human-Computer Interaction | 3 |
| 9 | BMJ Open | 2 |
| 10 | Children (Basel) | 2 |
| 11 | Clinical Pediatrics | 2 |
| 12 | Health Education Journal | 2 |
| 13 | International Journal of Environmental Research and Public Health | 2 |
| 14 | Italian Journal of Pediatrics | 2 |
| 15 | Italian Sociological Review | 2 |

***Note****.* The journals listed in this table belong to Zone 1 and are ordered by number of articles in descending order.

**Table S6. Most productive authors and their h-index and g-index values**

| **Author** | **Documents** | | **TC** | **h-index** | **g-index** | **m-index** | **NP** | **PY start** |
| --- | --- | --- | --- | --- | --- | --- | --- | --- |
| Cino D | | 6 | 67 | 6 | 6 | 0.86 | 6 | 2020 |
| Hong P | | 6 | 188 | 6 | 6 | 0.50 | 6 | 2015 |
| Chorney J | | 5 | 177 | 5 | 5 | 0.42 | 5 | 2015 |
| Fox A | | 4 | 137 | 4 | 4 | 0.50 | 4 | 2019 |
| Hoy M | | 4 | 137 | 4 | 4 | 0.50 | 4 | 2019 |
| Hudders L | | 4 | 38 | 2 | 4 | 0.67 | 4 | 2024 |
| Van D A E | | 4 | 38 | 2 | 4 | 0.67 | 4 | 2024 |
| Vanwesenbeeck I | | 4 | 38 | 2 | 4 | 0.67 | 4 | 2024 |
| Walrave M | | 4 | 128 | 4 | 4 | 0.50 | 4 | 2019 |
| Brosch A | | 3 | 151 | 2 | 3 | 0.18 | 3 | 2016 |
| Byford S | | 3 | 26 | 2 | 3 | 0.33 | 3 | 2021 |
| Goldsmith K | | 3 | 26 | 2 | 3 | 0.33 | 3 | 2021 |
| Hallam L | | 3 | 63 | 3 | 3 | 0.60 | 3 | 2022 |
| Kiliç S | | 3 | 9 | 2 | 3 | 0.67 | 3 | 2024 |
| Kostyrka-Allchorne K | | 3 | 26 | 2 | 3 | 0.33 | 3 | 2021 |
| Ritchie K | | 3 | 108 | 3 | 3 | 0.25 | 3 | 2015 |
| Romero-Rodríguez J | | 3 | 86 | 3 | 3 | 0.43 | 3 | 2020 |
| Shearer J | | 3 | 26 | 2 | 3 | 0.33 | 3 | 2021 |
| Sonuga-Barke E | | 3 | 26 | 2 | 3 | 0.33 | 3 | 2021 |
| Staes L | | 3 | 63 | 3 | 3 | 0.60 | 3 | 2022 |

***Note.*** Documents = number of publications in the corpus; TC = total citations; NP = number of cited publications included in the h-index calculation; PY start = first publication year; m-index = h-index divided by scientific age.

**Table S7. Characteristics of studies included in the metadata-coded narrative synthesis subset (n = 102)**

| **Dimension** | **Category** | **n** | **%** |
| --- | --- | --- | --- |
| Perspective | Parent-only empirical | 60 | 58.8 |
|  | Child/adolescent empirical | 5 | 4.9 |
|  | Mixed empirical | 3 | 2.9 |
|  | Conceptual/theoretical | 4 | 3.9 |
|  | Legal/policy | 23 | 22.5 |
|  | Review | 7 | 6.9 |
| Evidence type | Quantitative | 40 | 39.2 |
|  | Qualitative | 26 | 25.5 |
|  | Legal/policy analysis | 19 | 18.6 |
|  | Conceptual/commentary | 10 | 9.8 |
|  | Review | 7 | 6.9 |
| Age group represented in direct evidence | Parents only / no child sample | 94 | 92.2 |
|  | 14-17 | 6 | 5.9 |
|  | 10-13 | 1 | 1.0 |
|  | Mixed child/adolescent | 1 | 1.0 |
| Broad region | Europe | 62 | 60.8 |
|  | North America | 11 | 10.8 |
|  | Asia | 11 | 10.8 |
|  | Multi-region | 11 | 10.8 |
|  | Latin America | 3 | 2.9 |
|  | Oceania | 2 | 2.0 |
|  | Not clear | 2 | 2.0 |

***Note.*** Percentages are calculated within the coded narrative synthesis subset. Classifications were based on title, abstract, keywords, and source metadata. The supplementary numbering (S7-S8) was retained to avoid any confusion with table citations in the main manuscript.

**Table S8. Distribution of focal constructs by perspective in the metadata-coded narrative synthesis subset (n = 102)**

| **Perspective** | **n** | **Privacy** | **Autonomy** | **Digital identity** | **Consent** | **Online visibility** | **Boundary negotiation** | **Commercialization / influencer context** |
| --- | --- | --- | --- | --- | --- | --- | --- | --- |
| Parent-only empirical | 60 | 58 | 2 | 14 | 6 | 19 | 14 | 18 |
| Child/adolescent empirical | 5 | 4 | 2 | 2 | 1 | 1 | 0 | 0 |
| Mixed empirical | 3 | 3 | 0 | 0 | 1 | 0 | 2 | 0 |
| Conceptual/theoretical | 4 | 4 | 0 | 0 | 1 | 1 | 0 | 1 |
| Legal/policy | 23 | 20 | 5 | 6 | 6 | 3 | 1 | 4 |
| Review | 7 | 6 | 1 | 4 | 1 | 1 | 1 | 0 |

***Note.*** Counts across focal constructs are not mutually exclusive because a record could be coded under more than one construct. Classifications were based on title, abstract, keywords, and source metadata for the 102-record narrative synthesis subset.
